# Raven food calls indicate sender’s age and sex

**DOI:** 10.1186/s12983-018-0255-z

**Published:** 2018-03-13

**Authors:** Markus Boeckle, Georgine Szipl, Thomas Bugnyar

**Affiliations:** 1 Department of Cognitive Biology, University of Vienna, Vienna, Austria; 2 Konrad Lorenz Forschungsstelle, Core Facility, University of Vienna, Gruenau im Almtal, Austria; 3 Department of Psychology, University of Cambridge, Cambridge, UK

**Keywords:** *Corvus corax*, Raven, Food call, Sex, Age, Call production, Vocalization, Acoustic characteristics, Bioacoustics, Corvid

## Abstract

**Background:**

Acoustic parameters of animal signals have been shown to correlate with various phenotypic characteristics of the sender. These acoustic characteristics can be learned and categorized and thus are a basis for perceivers’ recognition abilities. One of the most demanding capacities is individual recognition, achievable only after repeated interactions with the same individual. Still, class-level recognition might be potentially important to perceivers who have not previously encountered callers but can classify unknown individuals according to the already learned categories. Especially for species with high fission-fusion dynamics that repeatedly encounter unknown individuals it may be advantageous to develop class-level recognition. We tested whether frequency-, temporal-, and amplitude-related acoustic parameters of vocalizations emitted by ravens, a species showing high fission-fusion dynamics in non-breeder aggregations, are connected to phenotypic characteristics and thus have the potential for class-level recognition.

**Results:**

The analysis of 418 food calls revealed that some components summarizing acoustic parameters were differentiated by age-classes and sex.

**Conclusions:**

Together, the results provide evidence for the co-variation of vocal characteristics and respective sex and age categories, a prerequisite for class-level recognition in perceivers. Perceivers that are ignorant of the caller’s identity can thus potentially recognize these class-level differences for decision-making processes in feeding contexts.

## Background

Territorial defence and parent-offspring are two well-studied contexts in which individual recognition is important [1–3], as individuals can benefit from memorizing the identity of the individuals as well as the type and/or outcome of previous interactions [4]. In species where individuals meet regularly after prolonged times, such as in fission-fusion systems [5], individual recognition based on acoustic communication appears highly beneficial [1, 5]. Still, in species with a high degree of fission-fusion dynamics, a large number of individuals within fluid groups, and large home-ranges, individuals might not only encounter known individuals but also unknown ones, for which no memory about previous interactions or information about their identity is available. During encounters with unknown individuals individual recognition is not possible, while class-level recognition can provide crucial benefits [1].

Vocal signals may convey various attributes of the vocalizer, among others sex (e.g.: [6]), age (e.g.: [7]), emotional state (e.g.: [8]), dominance rank hierarchy (e.g.: [9]), and reproductive status (e.g.: [10]). How and which of these attributes are obtained by perceivers from acoustic signals has been extensively studied in the last decades (e.g. [2]). Most of the acoustic parameters in focus relate to anatomical features of the producer and its production mechanisms. Fant [11] suggested that vocal production in humans is a two-stage process: vocalizations are produced by the vibrating tissue, and subsequently shaped by the vocal tract. This “source-filter theory” has successfully been generalized [12] to other mammals [13, 14], and also to birds [15–18]. In some species, vocal features such as fundamental frequency, frequency modulation, and other source dependent characteristics provide dependable cues to body size but also to genetic variation and age [19, 20].

In corvids like common ravens (*Corvus corax*), anatomic size differences between sexes and age groups are hypothesized to directly relate to acoustic parameters of the calls [21–25], providing cues to class-level recognition. Sex and age dependencies of acoustic signals in ravens might be similar to other bird species [26, 27] and seem linked to size differences between sexes and age-classes [28, 29]. Moreover, class-level distinction between sexes and age-classes that are based on acoustic features could be exploited by conspecifics for various forms of decision-making. In ravens, acoustic information about sex and age-class could be useful especially when encountering unknown individuals while deciding whether to engage in territorial defence or to join or avoid foraging groups [30–32]. Unlike features that are connected to caller identity [33], acoustic features related to sex, and age might not have to be learned of each single individual as cues to class-level recognition [1]. This is especially interesting in foraging ravens due to their fission-fusion dynamics [34–36]. Individuals gather at large and ephemeral carcasses, where they may encounter familiar and unfamiliar birds [37–41].

Ravens facing problems in accessing food are hypothesized to recruit conspecifics via vocalizations in order to reduce potential dangers and to overpower dominant conspecifics during feeding [30, 31, 37–41]. These food related calls, often referred to as ‘yells’ or long ‘haa’ calls [38, 40] are individually distinct [33]. While ravens were shown to discriminate between known and unknown individuals of different sexes, indicating class-level recognition [30], age-related differences in ‘haa’ calls have not been investigated, yet. Moreover, different age-classes have been described to differ in their food call characteristics [37, 40], but in-depth analysis of acoustic features is still missing. ‘Haa’ calls show highly harmonic structures in addition to non-linear phenomena in some calls. Resonance frequencies produced by the vocal tract, named formants, cannot be measured because of the highly harmonic structure of the calls as well as the fact that fundamental frequency and its harmonics differ around 800 Hz [33]. Due to large frequency ranges with no or low amplitude, any attributes in raven ‘haa’ calls that might indicate sex and age are primarily based on source related production mechanisms (i.e. fundamental frequency). We here investigate the variation of ‘haa’ call characteristics of common ravens related to age-classes and sex. We predict that in addition to previously described individual characteristics, age and sex differences are detectable in the food-related ‘haa’ call based on anatomical differences and potential variations in production mechanisms. In a society with high fission-fusion dynamics, known individuals might be recognized via individually distinct cues, while unknown callers could be classified according to age-class and sex, thereby assessing the degree of competition. Differentiating unknown individuals according to these class-specific cues can aid in decision-making processes, i.e. whether to approach or to retreat from an unfamiliar recruiting caller.

## Methods

### Study site and call recording

**Fig. 1:**
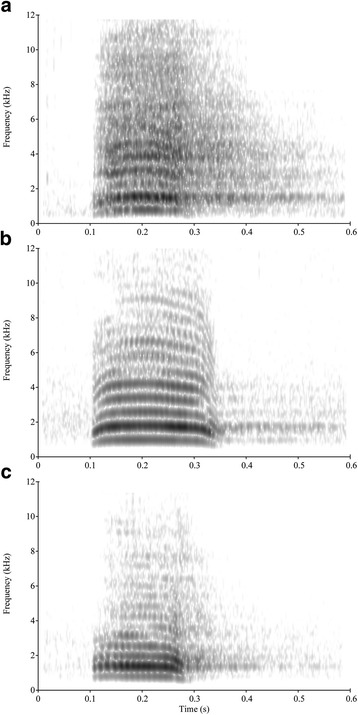
Spectrogram of a food call of (**a**) a juvenile, (**b**) a subadult, and (**c**) an adult common raven (FFT method, window length = 0.01, time step = 0.002, frequency step = 20, Gaussian shape)

Between summer 2009 and winter 2010 we recorded ‘haa’ calls of free-ranging common ravens that regularly forage inside the enclosures of the Cumberland Wildpark Grünau, Austria [42]. At the time of the study, approximately 100 ravens were marked individually with coloured leg bands and patagial wing tags. Individual information of these birds (e.g. weight, sex and age-class) was known [43]. Sex was genetically determined from blood samples (Laboklin, Austria). Age-classes were classified based on the coloration of the feathers and the inner beak: juveniles from fledging until the end of their first year have mostly pink oral cavities and brownish feathers; in subadults in their second and third year of life oral cavities turn from pink to black, i.e. are pinkish with dark speckles, and adults (> 3 years) have black oral cavities [44, 45]. The spectrograms in Fig. 1 show examples of ‘haa’ calls of each age-class.

We recorded calls of individually marked ravens between 0700 h and 0900 h at the feedings of wild boars (*Sus scrofa*), where ravens gather for foraging on a daily basis. We simultaneously video- and audio-recorded each feeding session to identify vocalizing individuals (Video-recorder: Canon HF-11 HD; microphone: Sennheiser ME67/K6; solid-state audio-recorder: Marantz PMD-670: sampling rate = 48 kHz, amplitude resolution = 16 bits) at distances ranging from 3 to 10 m. All calls with interfering background noise were removed. Additionally, we removed two individuals (two subadult males) represented with only one call each from the dataset, which provided us with 418 calls of 12 individuals (mean number of call per individual ± SD = 34.83 ± 34.51; 3 juveniles: 1 male(m), 2 females (f); 7 subadults: 2 m, 5 f; 7 adults: 1 m, 6 f).

### Call analysis

Acoustic analysis was conducted using a script in PRAAT 5.1.25 [46] that automatically logged acoustic variables in an output file. Because of the highly harmonic structure of the ‘haa’ call we mainly used source related acoustic features that are related to fundamental frequency (*f*o) using recently suggested terminology for acoustic measurements [47]. The analysed call parameters were mean *f*o (Hz), maximum *f*o (max *f*o; Hz), minimum *f*o (min *f*o; Hz), range of *f*o (Hz), start *f*o (Hz), end *f*o (Hz), *f*o at the half of call duration (mid *f*o; Hz), call duration (s), slope from *f*o start of the call to the *f*o maximum (slope S-M; Hz/s), slope from the maximum *f*o to the end of the call (slope M-E; Hz/s), inflection rate (number of frequency changes/s), harmonicity (HNR; dB), jitter (the absolute *f*o difference between consecutive *f*o measurements/the average period), *f*o variation (sum of all *f*o changes measured/call duration; Hz), amplitude range (maximum dB – minimum dB) and amplitude modulation (AM). All amplitude related measurements are independent of recording distance as they are relative measures within a call. Mean values of relevant call characteristics within age categories and sexes are listed in Table 1.

**Table 1:** Mean values and standard errors (SE) of acoustic variables used in the PCA

	Juvenile		Subadult		Adult	
	Female (*N* = 43;2)	Male (*N* = 6;1)	Female (*N* = 137;5)	Male (*N* = 27;2)	Female (*N* = 198;6)	Male (*N* = 7,1)
	Mean ± SE	Mean ± SE	Mean ± SE	Mean ± SE	Mean ± SE	Mean ± SE
Mean *f*o (Hz)	755.053 ± 15.527	774.468 ± 3.277	679.941 ± 7.605	715.327 ± 6.215	643.749 ± 3.196	598.14 ± 2.421
Maximum *f*o (Hz)	809.845 ± 12.933	827.117 ± 4.627	709.005 ± 7.953	755.367 ± 6.373	671.995 ± 3.523	619.023 ± 3.119
Start *f*o (Hz)	658.953 ± 17.434	647.698 ± 9.471	640.394 ± 7.155	626.777 ± 8.569	595.298 ± 2.976	578.75 ± 6.946
Mid *f*o (Hz)	793.836 ± 16.825	823.3 ± 4.254	702.317 ± 8.131	751.162 ± 6.753	666.727 ± 3.674	616.813 ± 3.018
Call duration (s)	0.299 ± 0.014	0.237 ± 0.006	0.217 ± 0.003	0.226 ± 0.004	0.201 ± 0.002	0.2 ± 0.003
HNR (dB)	10.587 ± 0.788	19.338 ± 0.228	13.916 ± 0.279	15.819 ± 0.549	16.055 ± 0.186	21.36 ± 0.296
Jitter	0.035 ± 0.003	0.01 ± 0	0.014 ± 0.001	0.013 ± 0.001	0.011 ± 0	0.01 ± 0
Amplitude modulation	32.034 ± 1.436	30.058 ± 2.225	39.325 ± 1.919	30.413 ± 1.235	30.921 ± 0.773	30.579 ± 2.221
Amplitude range (dB)	9.422 ± 0.553	9.352 ± 0.656	8.724 ± 0.397	10.51 ± 1.008	8.615 ± 0.213	12.571 ± 1.116

N denotes the number of calls analysed; and the number of individuals per age-class and sex. Five individuals were sampled in two age-classes

### Statistical analysis

A Principal Component Analysis (PCA) was conducted to reduce the amount of acoustic variables after partially correlated variables were removed. Three Principal Components (PCs) were extracted with an eigenvalue greater than 1.0 using a varimax rotation.

Three linear mixed-effect models (LMMs) were calculated using the PC scores as response variables. Individual identity was entered as a random effect to account for repeated sampling. As potential fixed effects, sex, age-class, and weight at trapping were tested for multicollinearity by calculating Variance Inflation Factors (VIF) [48]. Sex and weight showed high collinearity, and thus weight was investigated separately using nonparametric Spearman rank correlations. As fixed effects in the LMMs, sex and age-class were used. For model selection (Table 2) models were ranked by their differences in AICc (ΔAICc), that were calculated by subtracting the lowest AICc from all other AICc values. The relative likelihoods (exp (− 0.5/ΔAICc)) and Akaike weights (relative likelihood/sum of all relative likelihoods) were computed as measures of strength of evidence for each model [49]. When several models had high support (Δi ≤ 2), model averaging was conducted (Table 3). In order to obtain all coefficients in the comparision between juveniles, subadults and adults we changed the reference category an reran the models. Estimated mean values, z and *p* values were obtained from the averaged models for all coefficients.

**Table 2:** Model selection for the LMMs investigating the effects of sex and age-class on for the three Principle Components (PC1-PC3)

Models	AICc	Δi	Relative likelihood	Akaike weight
PC1				
Sex + Age-class	614.59	4.80	0.091	0.053
**Sex**	**611.67**	**1.89**	**0.388**	**0.227**
Age-class	612.69	2.91	0.234	0.137
**Null**	**609.78**	**0.00**	**1.000**	**0.584**
PC2				
**Sex + Age-class**	**637.81**	**1.69**	**0.429**	**0.300**
Sex	652.81	16.69	0.000	0.000
**Age-class**	**636.12**	**0.00**	**1.000**	**0.699**
Null	650.96	14.84	0.001	0.000
PC3				
**Sex + Age-class**	**1076.32**	**1.04**	**0.595**	**0.373**
Sex	1116.96	41.68	0.000	0.000
**Age-class**	**1075.29**	**0.00**	**1.000**	**0.627**
Null	1118.62	43.34	0.000	0.000

Corrected Akaike Information Criterion (AICc) values, their differences (Δi), the relative likelihood, and the resulting Akaike weights are shown for each model. Models with highest support (Δi≦ 2) are indicated in bold type

**Table 3:** Averaged LMMs investigating the effects of sex and age-class onto the three Principal Components (PC1-PC3), with coefficients, estimated means (EM), standard error (SE), *z* values, significances (p), and lower and upper confidence intervals (CI)

Coefficients	EM	SE	*z* value	p	2.5% CI	97.5% CI
PC1						
(Intercept)	− 0.18	0.29	0.62	0.537	− 0.74	0.39
Sex^a^ (female vs. male)	0.24	0.63	0.38	0.701	− 0.99	1.48
PC2						
(Intercept)	0.51	0.34	1.48	0.1380	− 0.16	1.19
Sex^a^ (female vs. male)	− 0.43	0.71	0.61	0.5424	−1.81	0.95
Age-class^b^ (juvenile vs. subadult)	− 0.06	0.12	0.50	0.6195	− 0.29	0.17
Age-class^b^ (juvenile vs. adult)	− 0.43	0.14	2.96	0.0031	− 0.71	− 0.14
Age-class^c^ (subadult vs. adult)	− 0.37	0.08	4.37	< 0.0001	− 0.53	− 0.20
PC3						
(Intercept)	1.40	0.34	4.11	0.0000	0.73	2.07
Sex^a^ (female vs. male)	0.62	0.60	1.03	0.3021	− 0.55	1.78
Age-class^b^ (juvenile vs. subadult)	− 0.76	0.20	3.80	0.0001	−1.15	− 0.37
Age-class^b^ (juvenile vs. adult)	−1.63	0.24	6.77	< 0.0001	−2.10	−1.16
Age-class^c^ (subadult vs. adult)	− 0.87	0.14	6.16	< 0.0001	−1.15	− 0.60

Set as reference point: ^a^female, ^b^juvenile, ^c^subadult

Statistical analysis was performed in R Version 3.3.3 [50] using the packages GPA rotation (version 2014.11–1 [51]), psych (version 1.7.3.21 [52]), AICcmodavg [53], MuMIn (version 1.15.6 [54]), and lme4 (version 1.1–13 [55]). Estimated values, confidence intervals (CI) as well as z- and *p*-values were calculated with functions in the package MuMIn (version 1.15.6 [54]).

## Results

Principle component analysis resulted in three factors. The three PCs explained 81% of the overall variance. Measures of *f*o (mean *f*o, maximum *f*o, start and mid *f*o) loaded on the first PC and explained 41% of the variance. PC2 contained the acoustic variables call duration, HNR, and jitter, and contributed 27% to the overall variance. Amplitude-related vocal parameters (amplitude modulation and amplitude range) loaded on the third PC and explained 14% of the variance. The standardized loadings are shown in Table 2. PC scores were extracted for further analyses.

**Fig. 2:**
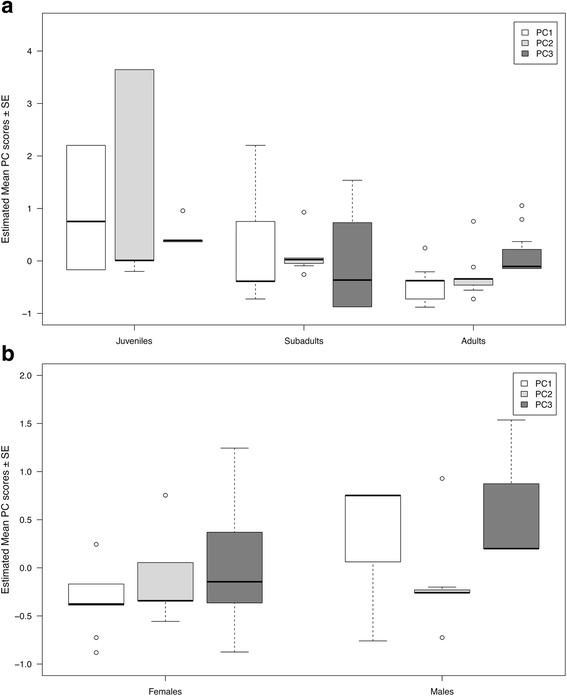
Estimated means ± standard errors (SE) of the three PCs for different age-classes (**a**), and for male and female common ravens (**b**). PC1 summarizes acoustic properties related to the fundamental frequency of “haa” calls, PC2 includes call duration and source-related acoustic features, and PC3 amplitude-related measures

Model selection procedure revealed that in PC1 the null-model as well as the model including sex explains most of the variance (Table 4); in PC2 and PC3 the models with either age-class alone or with sex and age-class explained the variance the best. Scores of PC2 were highest in juveniles and decreased in subadults, and were lowest adults (Table 3, Fig. 2a). Scores of PC3 were also found to be highest in juveniles and lower in subadults, but higher in adults as compared to subadult individuals (Table 4, Fig. 2a). Sex did not show a strong influence on the regression scores of PC2 and PC3 (cp. Fig. 2b). Scores of PC1 did not vary with age-class or sex (Table 4, Fig. 2a and b).

**Table 4:** Component matrix of the PCA with loadings of each acoustic variable

Acoustic Variable	Principal Components		
	1	2	3
Mean *f*o	**0.99**	0.08	− 0.03
Maximum *f*o	**0.97**	0.18	0.00
Start *f*o	**0.84**	− 0.14	− 0.16
Mid *f*o	**0.97**	0.12	0.00
Call duration	0.23	**0.87**	− 0.03
HNR	0.10	**− 0.80**	0.36
Jitter	0.04	**0.94**	0.03
Amplitude modulation	− 0.03	0.14	**− 0.70**
Amplitude range	− 0.16	− 0.01	**0.81**

The dimension of the acoustic variables was reduced to three Principal Components (KMO = 0.73). Interpretable factor loadings are indicated in bold

There was no significant correlation between weight and the scores of PC1 (Spearman rank correlation: rs = − 0.021, *p* = 0.9562), PC2 (rs = − 1.175, *p* = 0.5883), and PC3 (rs = 0.538, *p* = 0.0749).

## Discussion

We here showed that food-associated ‘haa’ calls of ravens disclose sex- and age-related characteristics about the phenotype of the caller. These results support the idea of class-specific cues in acoustic signals that would enable class-level recognition [1], i.e. that naïve ravens listening to ‘haa’ calls may extract information about the age-class and the sex of the callers.

Differentiating unknown callers in a social system with high degrees of fission-fusion helps in decision-making processes, when individual recognition is not possible based on missing knowledge on about others. In ravens, vast numbers of individuals gather for roosting [56–58] and feeding [30, 31, 37, 38, 41]. The numbers of individuals within a group fluctuate throughout the year while the stratification of the group based on relationship qualities according to sex, age, and kinship stays consistent [43, 59, 60]. These constantly changing groups impose high demands and challenges on each individual when evaluating collaborative and competitive interests for large numbers of conspecifics. Common ravens show collaborations in feeding situations via recruitment [40, 56–58] but at the same time compete for resources. As aggression during foraging in ravens is relatively high, and fights could cause costly injuries, decisions about whether to join or avoid a feeding situation can be crucial. By assessing acoustic cues about sex and age, the relative strength and reliability of unknown ‘haa’ callers are conveyed in addition to food-availability [30, 38, 40, 41].

The reliability of recruitment to food in ravens increases with age [37], and thus perceivers might be able to assess signal reliability based on callers’ age. The ability of perceivers to selectively respond to specific classes has been reported for instance in alarm calls of marmots (*Marmota flaviventris*) where juvenile calls elicit more attention [61], and in vervet monkey calls (*Cercopithecus aethiops*), where the reliability of the signaller was learned in a playback study [62]. Caller reliability appears highly crucial for the evolution and maintenance of alarm call and food call communication [63]. Additionally, juvenile senders of food-associated calls might profit from indicating their age to unknown conspecifics. As juvenile food-associated calls (also termed ‘chii calls’) are supposed to derive from begging calls [40], these calls may indicate parents about the hunger level of their offspring [40] and might function as puppy licence. Thus, perceivers of these calls might take into account that parent ravens could be in the vicinity and defend their young.

It is noteworthy that, compared to females, males tend to show low rates of food-associated calls [31]. In a previous experiment, where raven food-associated calls were played back in the wild, nine out of ten birds responded to females [30]. As females are in general lower in rank [64], especially higher ranking males might profit from approaching food-calling females. A similar effect was found in brown capuchin monkeys (*Cebus apella*), where lower-ranking females call more than higher-ranking individuals [65]. Additionally, low-ranking ravens might benefit from attracting other non-breeders especially when calling within a territory of a breeding raven pair. By increasing the number of non-breeders and thus overpowering the territorial pair, food accessibility might be secured. Furthermore, dominant male callers may use another food-related call (‘who’; [37]). This call type might indicate different phenotypic information than the here presented ‘haa’ calls.

PC1, which combined acoustic variables related to *f*o, showed least evidence for explaining sex and age-class related differences. Still, differences in PC1 do exist and were previously related to individual recognition [33]. They could be size dependent, as after fledging and gaining weight, developmental changes of internal structures like ossification of tracheal and syringeal cartilaginous rings take place, and thus can cause changes in *f*o due to anatomical changes of the syrinx like size post-fledging [66]. Additionally, neural changes due to the ontogenetic development of the caller might correlate with our classification of age-classes that potentially relate to individuality. Neural changes, like increases of the HVC after sexual maturing [67], might be reflected in differences of *f*o.

Furthermore, deterministic chaos, which is reflected in HNR of a call, was included in PC2. It is important in the acoustic communication of animals [68, 69] as it can signal urgency or motivation (e.g. baby cries [70], monkey alarm calls [71]), and might be perceived by listening individuals (e.g.: [72, 73]). In relation to the ontogenetic development of the individual we expect a decrease of urgency-related features in ‘haa’-calls that might also relate to the motivation [8] i.e. hunger level of the caller. In congruence with this motivation-structural rule, food-associated calls are hypothesized to develop from begging calls [40]. In addition to HNR also jitter is included in PC2. Mammals are known to increase jitter based on changes of oestrogen in females [19, 74] and of testosterone in males [19, 75]. Similar mechanisms based on hormonal changes could be at play in raven ‘haa’ calls that might relate to urgency of the callers. Similarly, call duration is represented in PC2 and is often related to urgency [76]. Highest levels are found in juvenile females bearing the lowest rank in raven societies and thus might encounter high levels of constrains in gaining access to food [64].

Amplitude modulation is mainly represented in PC3 and varies according to age-class. We suggest that similar to deterministic chaos, jitter, and call duration, an increase of amplitude modulation is related to urgency. Still, amplitude modulation has not been considered in many animals and was considered as low hierarchy parameter, i.e. transmitting little information [77].

As male ravens are in general larger than females, gross body mass and size differences [28] might correlate with differences in syringeal structures, and cause sexually dimorphic acoustic features of raven calls. Such size-related differences have been reported for jungle crows [22] and other bird species like murres [78], while to our knowledge no such differences were reported in the literature for ravens, yet. Despite this effect, it has been discussed that based on small effect sizes, *f*o differences have a low reliability as indicators of body size in birds [79]. Hence, *f*o variances might not be good indicators for the sex of the calling raven when sex differences are merely based on size-dependent differences in syringeal anatomy. Note that the weight of the studied individuals did not correlate with either of the PCs, confirming previously shown small effect sizes.

Similarly, differences in hormone levels between male and female birds can cause variation in calling behaviour [80] and activity in the neural song control regions [81]. While hormonal changes have been shown to affect the vibrational properties of sound-producing structures in mammals [74] to our knowledge such an effect has not been documented for birds. Especially bird species with monomorphic singing and calling behaviour are less studied [82] in their differences in higher vocal centre (HVC) structures. Still, sexually dimorphic neural structures might cause sexually dimorphic calls in ravens, which has to be studied in more detail.

Measures of amplitude modulation and range cluster in PC3 and relate to age-classes. While most of the measures decrease or increase with age, amplitude-related parameters are lower in subadults than in adults. This effect also is in contrast to reduced variation in all parameters with increasing age (see Table 3) and might be connected to morphological changes during maturation. Age classification of ‘haa’ calls is strongly supported by our data, especially based on acoustic variables in PC2 and PC3. We hypothesize that labial flexibility, mass, and length, which have been shown to vary with age in mammals [83], might change as signallers mature. Structural differences of the vocalizing apparatus in turn determine acoustic features of a vocalization (e.g. [83]). In addition to variations in the vocal organ, maturation of neural structures based on testosterone-induced growth of the HVC [84] might additionally influence acoustic features of raven calls, as was shown in birdsong [85].

## Conclusion

Taken together, we herewith show that raven ‘haa’ calls vary according to sex and age of the vocalizer and might be the underlying mechanism of class-level recognition. Especially in food-related calls that are recruiting conspecifics to potentially dangerous feeding situations, class-level recognition could help when encountering unknown individuals while individual recognition is used during repeated interactions with already known individuals. Thus, ravens with a pronounced level of fission-fusion seem to possibly make use of class-level recognition as well as individual recognition during their complex feeding behaviour.
